# Comparing the Efficacy and Long-Term Outcomes of Sodium-Glucose Cotransporter-2 (SGLT2) Inhibitors, Dipeptidyl Peptidase-4 (DPP-4) Inhibitors, Metformin, and Insulin in the Management of Type 2 Diabetes Mellitus

**DOI:** 10.7759/cureus.74400

**Published:** 2024-11-25

**Authors:** Farhan Khan, Tanjil Hussain, Taha Zahid Chaudhry, FNU Payal, Abdullah Shehryar, Abdur Rehman, Afif Ramadhan, Muhammad Tassaduq Hayat, Muath M Dabas, Mustafa Khan

**Affiliations:** 1 Internal Medicine, Rehman Medical Institute, Peshawar, PAK; 2 Internal Medicine, London North West Hospitals NHS Trust, London, GBR; 3 Internal Medicine, Holy Family Hospital, Rawalpindi, PAK; 4 Internal Medicine, Ghulam Muhammad Mahar Medical College, Karachi, PAK; 5 Internal Medicine, Allama Iqbal Medical College, Lahore, PAK; 6 Surgery, Mayo Hospital, Lahore, PAK; 7 Internal Medicine, Gadjah Mada University, Yogyakarta, IDN; 8 Internal Medicine, Chandka Medical College, Larkana, PAK; 9 Internal Medicine, Shaheed Mohtarma Benazir Bhutto Medical University, Larkana, PAK; 10 Surgery, The University of Jordan, Amman, JOR; 11 General Surgery, Nishtar Medical University, Multan, PAK

**Keywords:** cardiovascular outcomes, dpp-4 inhibitors, glycemic control, hypoglycemia risk, insulin, metformin, renal protection, sglt2 inhibitors, type 2 diabetes mellitus, weight changes

## Abstract

Type 2 diabetes mellitus (T2DM) is a chronic metabolic disorder characterized by hyperglycemia, insulin resistance, and decreased insulin secretion. With its rising global prevalence, effective management strategies are critical to reducing morbidity and mortality. This systematic review compares the efficacy, safety, and long-term outcomes of four major pharmacological treatments for T2DM: sodium-glucose cotransporter-2 (SGLT2) inhibitors, dipeptidyl peptidase-4 (DPP-4) inhibitors, metformin, and insulin. We focused on randomized controlled trials (RCTs) published within the last five years (2019-2024) to provide an up-to-date assessment of glycemic control, cardiovascular and renal benefits, weight effects, and the risk of hypoglycemia. The review highlights that while all four medication classes effectively reduce HbA1c levels, SGLT2 inhibitors stand out for their additional cardiovascular and renal benefits, including significant reductions in major adverse cardiovascular events and chronic kidney disease progression. Metformin remains a cornerstone first-line therapy due to its safety, efficacy, and affordability. DPP-4 inhibitors are a weight-neutral, well-tolerated option, although their efficacy may diminish over time. Insulin, while the most potent glucose-lowering agent, carries a higher risk of hypoglycemia and weight gain. Our findings emphasize the importance of personalized, patient-centered approaches that account for the distinct therapeutic profiles of these treatments. Future research should prioritize head-to-head comparisons and optimal therapy sequencing to refine treatment guidelines for diverse patient populations.

## Introduction and background

Type 2 diabetes mellitus (T2DM) is a chronic metabolic disorder characterized by hyperglycemia resulting from insulin resistance and/or decreased insulin secretion [1]. The global prevalence of T2DM has risen dramatically in recent decades, with an estimated 462 million individuals affected worldwide in 2017, representing 6.28% of the global population [2]. This escalating prevalence, coupled with the significant morbidity and mortality associated with T2DM, underscores the critical importance of effective management strategies.

The management of T2DM has evolved significantly over the past few decades, with the introduction of several new classes of glucose-lowering medications. While metformin remains widely regarded as the first-line pharmacological treatment for most patients with T2DM [3], its positioning as a universal first-line therapy continues to be evaluated in light of emerging evidence and individual patient needs. Among the newer classes of medications, sodium-glucose cotransporter-2 (SGLT2) inhibitors and dipeptidyl peptidase-4 (DPP-4) inhibitors have gained increasing prominence. These agents have demonstrated significant efficacy in glycemic control and are generally well-tolerated, with favorable safety profiles in appropriate patient populations [4,5].

SGLT2 inhibitors work by reducing renal glucose reabsorption, leading to increased urinary glucose excretion [6]. DPP-4 inhibitors, on the other hand, increase the levels of incretin hormones, which stimulate insulin secretion and suppress glucagon release [7]. These mechanisms are distinct from those of metformin, which primarily reduces hepatic glucose production and improves insulin sensitivity [8], and insulin, which directly lowers blood glucose levels by facilitating glucose uptake in peripheral tissues [9].

While each of these medication classes has demonstrated efficacy in glycemic control, their comparative effectiveness, particularly in terms of long-term outcomes, remains an area of active investigation. Factors such as cardiovascular benefits, renal protection, weight effects, and hypoglycemia risk vary among these treatments and can significantly impact patient outcomes and quality of life [10].

The objective of this comprehensive analysis is to evaluate and compare the efficacy, safety, and long-term outcomes of SGLT2 inhibitors, DPP-4 inhibitors, metformin, and insulin in the management of T2DM. By synthesizing findings from various clinical studies, we aim to provide an evidence-based assessment that can contribute to the refinement of treatment algorithms. This analysis is intended to support healthcare providers in making more informed, personalized treatment decisions, ultimately improving patient outcomes by considering key factors such as glycemic control, cardiovascular and renal benefits, weight effects, and the risk of hypoglycemia. Additionally, this review seeks to highlight the role of combination therapies in optimizing the management of T2DM, particularly for diverse patient populations with complex needs.

## Review

Materials and methods

Search Strategy

Our search strategy followed the Preferred Reporting Items for Systematic Reviews and Meta-Analyses (PRISMA) guidelines to identify randomized controlled trials (RCTs) published within the last five years (2019-2024). Searches were conducted across several major electronic databases, including PubMed, Medline, Embase, the Cochrane Library, and CINAHL, up to September 2024.

We utilized a combination of Medical Subject Headings (MeSH) terms and keywords such as "Type 2 Diabetes Mellitus", "SGLT2 inhibitors", "DPP-4 inhibitors", "Metformin", "Insulin", and "combination therapy". Boolean operators ("AND", "OR") were applied to refine the search queries. Examples of search terms included: "Type 2 Diabetes AND SGLT2 inhibitors AND DPP-4 inhibitors", "Metformin AND Insulin AND glycemic control", and "combination therapy AND long-term outcomes". In addition to database searches, the reference lists of selected articles were manually screened to identify any further relevant studies. We limited our search to studies published in English and peer-reviewed journals.

Eligibility Criteria

Our inclusion criteria focused exclusively on RCTs published within the last five years (2019-2024), investigating the efficacy, safety, and long-term outcomes of SGLT2 inhibitors, DPP-4 inhibitors, metformin, and insulin, either as monotherapy or combination therapy, in patients with T2DM. Studies were required to report key outcomes such as glycemic control (HbA1c), cardiovascular events, renal outcomes, body weight changes, and hypoglycemia incidence. Studies that did not meet these criteria were excluded, including non-RCTs, studies published prior to 2019, animal studies, non-peer-reviewed literature, and those focusing on non-adult or gestational diabetes populations. Additionally, we excluded studies that did not provide sufficient data on the primary outcomes of interest.

Data Extraction

Two independent reviewers conducted the screening of titles and abstracts to ensure relevance based on our inclusion criteria. Full-text reviews were then performed for studies deemed potentially eligible. Data extraction was conducted using a standardized form in Microsoft Excel (Microsoft Corporation, Redmond, Washington), capturing information such as study design, sample size, patient characteristics, treatment regimens, and outcomes. In cases where there were disagreements between the reviewers, a third reviewer resolved the discrepancies to ensure consistency and accuracy in the selection process.

Data Analysis and Synthesis

Given the variability in study design and reported outcomes, we performed a narrative synthesis rather than a meta-analysis. Studies were categorized based on key outcomes, including glycemic control, cardiovascular and renal outcomes, weight changes, and hypoglycemia risks. The synthesis identified common patterns and notable differences between the treatment modalities, particularly in terms of long-term efficacy and safety. Our narrative analysis provides a holistic assessment of the relative benefits and risks of these therapies, with the aim of informing evidence-based clinical decision-making in the management of T2DM.

Results

Study Selection Process

**Figure 1:**
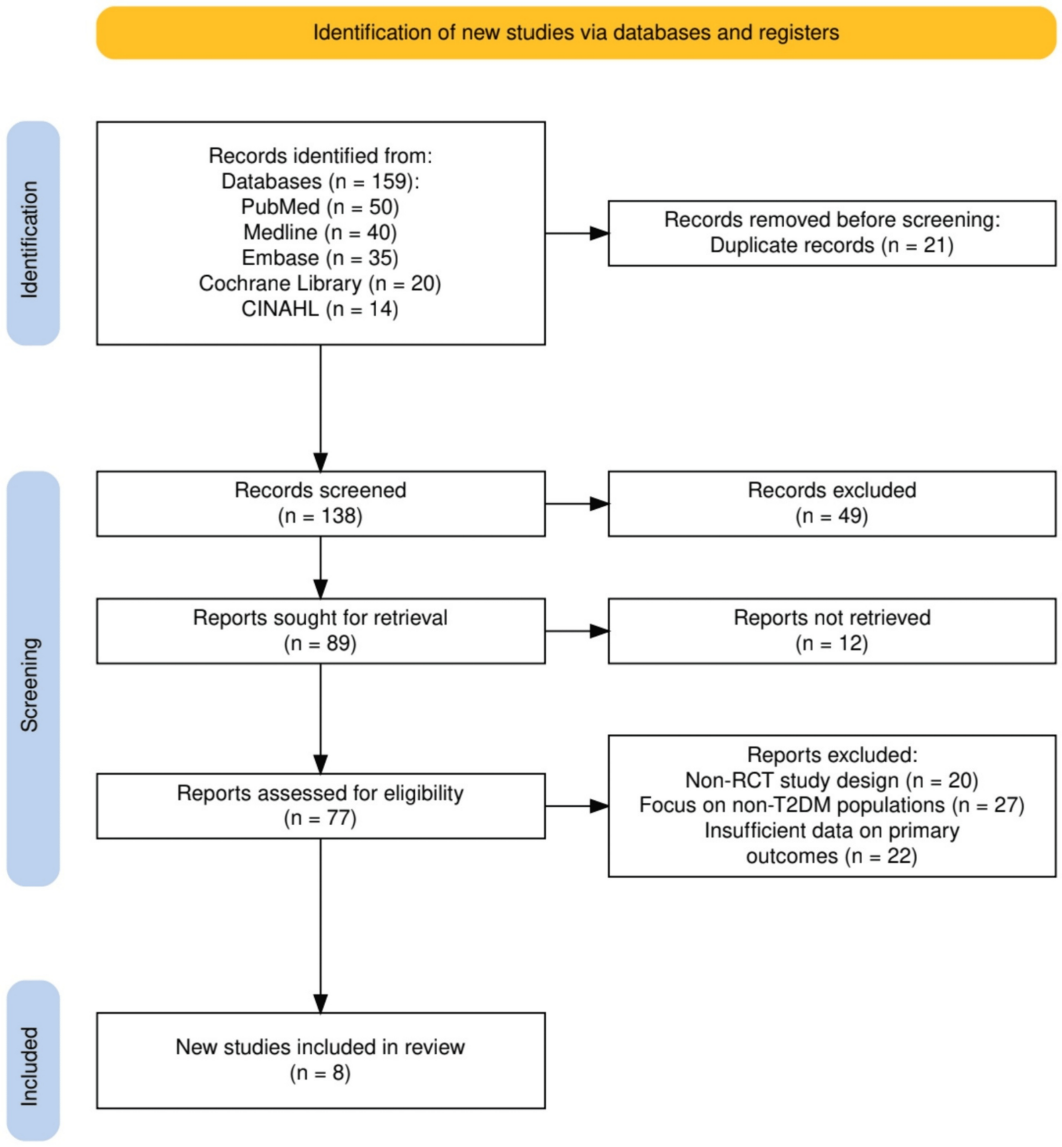
The PRISMA flowchart represents the study selection process. PRISMA: Preferred Reporting Items for Systematic Reviews and Meta-Analyses

A total of 159 studies were identified from various databases, including PubMed, Medline, Embase, the Cochrane Library, and CINAHL. After removing 21 duplicate records, 138 studies were screened based on titles and abstracts. During the screening process, 49 studies were excluded for not meeting the eligibility criteria. The full text of 89 studies was sought for further review, but 12 reports could not be retrieved. Subsequently, 77 studies were assessed for eligibility. Of these, 69 studies were excluded for reasons such as non-RCT study designs (20 studies), focusing on non-T2DM populations (27 studies), or providing insufficient data on primary outcomes (22 studies). Ultimately, eight studies met all the inclusion criteria and were included in the final review. The study selection process is given in Figure 1.

Characteristics of the Selected Studies

The selected studies for this review include a range of RCTs that explore the efficacy of various pharmacological treatments for T2DM. Across the trials, different combinations of SGLT2 inhibitors, DPP-4 inhibitors, metformin, and insulin were administered to evaluate their impact on glycemic control, cardiovascular and renal outcomes, weight changes, and hypoglycemia risks. The primary outcomes in most studies were changes in HbA1c levels, with secondary outcomes including reductions in fasting plasma glucose, body weight, and blood pressure. Key findings highlighted that combination therapies involving SGLT2 inhibitors and DPP-4 inhibitors often resulted in greater reductions in HbA1c and weight loss compared to other treatments, while insulin was more effective in lowering glucose but came with a higher risk of hypoglycemia and weight gain. Additionally, SGLT2 inhibitors demonstrated significant cardiovascular and renal benefits, particularly in patients with existing comorbidities. The characteristics of the selected studies are presented in Table 1.

**Table 1:** Characteristics of the key studies. HbA1c: Hemoglobin A1c; FDC: Fixed-Dose Combination; DAPA: Dapagliflozin; SITA: Sitagliptin; MET ER: Metformin Extended Release; MET SR: Metformin Sustained Release; T2D: Type 2 Diabetes; BMI: Body Mass Index; DAPA+SAXA: Dapagliflozin + Saxagliptin; GLIM: Glimepiride; MRI: Magnetic Resonance Imaging; TCT: Triple Combination Therapy (Metformin, Dapagliflozin, Saxagliptin); SAT: Sequential Add-on Therapy (Metformin, Glimepiride, Sitagliptin); INS: Insulin; T2DM: Type 2 Diabetes Mellitus; HDL: High-Density Lipoprotein

Article	Study	Study Design	Participants	Interventions	Primary Outcome	Key Findings
1	Laffel LM et al., 2023 [11]	Multicenter, randomized, double-blind, parallel-group, phase 3 trial	262 screened, 158 randomly assigned; aged 10-17 with type 2 diabetes previously treated with metformin or insulin	1:1:1 randomization to oral empagliflozin 10 mg, oral linagliptin 5 mg, or placebo. Secondary randomizations for dosage adjustments at weeks 12 and 26	Change from baseline in HbA1c at 26 weeks	At week 26, empagliflozin showed a significant reduction in HbA1c (-0.84%) compared to placebo (p=0.012); linagliptin did not show a significant change compared to placebo (-0.34%, p=0.29).
2	Sahay RK et al., 2023 [12]	Phase 3, randomized, open-label, active-controlled study	Adult patients with HbA1c ≥ 8% and ≤ 11%, randomized in 1:1:1 ratio to receive different drug combinations (n=415 total)	FDC of DAPA + SITA + MET ER (10 mg + 100 mg + 1000 mg) vs. SITA + MET SR (100 mg + 1000 mg) vs. DAPA + MET ER (10 mg + 1000 mg), all once daily	Mean change in HbA1c from baseline to week 16	Triple combination DAPA + SITA + MET ER showed significantly greater HbA1c reduction compared to both dual combinations. Notable postprandial and fasting glucose reductions were also observed.
3	Frías JP et al., 2022 [13]	104-week extension of a 52-week global, multicenter, parallel-group, active-controlled, double-blind study	Adult participants with T2D (HbA1c 58.5-91.3 mmol/mol (7.5%-10.5%)) and BMI 20.0 to 45.0 kg/m^2^, receiving MET (≥1500 mg/d), 382 entered, 338 completed the study	DAPA+SAXA (10/5 mg) vs. GLIM (1-6 mg), both plus placebo, once daily	Requirement for treatment intensification based on HbA1c, achieving therapeutic glycaemic response, changes in adipose tissue and liver fat on MRI	Lower treatment intensification is needed with DAPA+SAXA+MET. Greater reduction in liver fat and adipose volumes. A higher percentage achieved therapeutic glycaemic response with DAPA+SAXA+MET.
4	Kim NH et al., 2024 [14]	Multicenter, randomized, 104-week, open-label, active-controlled trial	105 drug-naïve patients with T2D (HbA1c level ≥ 8.0%, < 11.0%)	TCT (1000 mg of metformin, 10 mg of dapagliflozin, and 5 mg of saxagliptin once daily) vs. SAT (initiated with metformin, followed by glimepiride and sitagliptin)	The proportion of patients achieving HbA1c < 6.5% without hypoglycemia, significant weight gain, or drug discontinuation at week 104	TCT showed similar HbA1c reduction but a significantly higher achievement of primary outcome vs. SAT. Lower incidence of hypoglycemia, weight gain, and drug discontinuation in the TCT group.
5	Vilsbøll T et al., 2020 [15]	International Phase 3 study, randomized, parallel-design, open-label	1163 patients with T2D on metformin with/without sulphonylurea, 643 received treatment, 600 entered the long-term phase	DAPA + SAXA vs. INS for 24 weeks (short-term) with a 28-week (long-term) extension	Adjusted mean change from baseline in HbA1c and body weight at week 52; proportion achieving optimal glycaemic response without hypoglycemia and without requiring rescue medication	DAPA + SAXA resulted in greater HbA1c reduction and weight loss compared to INS. A higher proportion achieved HbA1c <7.0% without hypoglycemia in the DAPA + SAXA group.
6	Liu S-C et al., 2021 [16]	24-week, open-label, parallel-design randomized controlled trial	Patients with poorly controlled T2DM despite a premixed insulin regimen (n=106)	5mg of linagliptin (n=53) vs. 25mg of empagliflozin (n=53) for 24 weeks	Change in glycated hemoglobin (HbA1c) from baseline at week 24	The Empagliflozin group showed significant reductions in HbA1c, fasting plasma glucose, body weight, systolic blood pressure, and daily insulin dose compared to the linagliptin group.
7	Frias JP et al., 2020 [17]	52-week, multicenter, double-blind, active-controlled study	Patients inadequately controlled on metformin (n=444), randomized to DAPA + SAXA (n=227) or GLIM (n=217)	DAPA 10 mg + SAXA 5 mg vs. GLIM 1-6 mg (titrated)	Change in HbA1c from baseline to week 52	DAPA + SAXA showed greater reductions in HbA1c, body weight, and systolic blood pressure compared to GLIM. A higher proportion of patients achieved HbA1c <7.0%, with fewer requiring treatment intensification.
8	Fuchigami A et al., 2020 [18]	Prospective, randomized, open-label, blinded-endpoint, parallel-group trial	340 Japanese patients with early-stage T2D receiving metformin alone or no glucose-lowering agents	Dapagliflozin vs. sitagliptin for 24 weeks	Proportion of patients achieving HbA1c < 7.0%, avoidance of hypoglycemia, and ≥ 3.0% body weight loss	The Dapagliflozin group achieved a higher overall primary endpoint than sitagliptin. Greater reductions in body weight, fasting plasma glucose, and uric acid. It improved HDL cholesterol and stabilized renal functions.

Discussion

This systematic review offers a comprehensive and detailed comparison of the efficacy and long-term outcomes of four major pharmacological treatments (SGLT2 inhibitors, DPP-4 inhibitors, metformin, and insulin) in the management of T2DM. By synthesizing the most recent evidence from RCTs, our review provides valuable insights into the distinct therapeutic profiles of these medications. SGLT2 inhibitors, for instance, have demonstrated not only robust glycemic control but also substantial cardiovascular and renal benefits, making them particularly suitable for patients with comorbidities such as heart failure or chronic kidney disease. DPP-4 inhibitors, while effective in lowering blood glucose, present a more neutral cardiovascular profile, which can be advantageous in patients who are at risk for adverse cardiovascular events but cannot tolerate other, more aggressive treatments. Metformin remains a cornerstone therapy due to its efficacy, safety, and cost-effectiveness, alongside its modest cardiovascular benefits. Conversely, insulin is the most potent in controlling hyperglycemia but carries a higher risk of hypoglycemia and weight gain, which may limit its use in certain populations. Our findings emphasize the importance of personalized treatment approaches, considering the unique clinical profiles of patients, including their cardiovascular risk, renal function, and potential for hypoglycemia. These insights contribute to the ongoing refinement of T2DM management guidelines by highlighting the relative benefits and risks of each treatment option, thus supporting more informed, patient-centered clinical decision-making.

Our analysis reveals that all four medication classes (SGLT2 inhibitors, DPP-4 inhibitors, metformin, and insulin) demonstrate significant efficacy in improving glycemic control, as measured by reductions in HbA1c levels. Consistent with previous studies, metformin remains a highly effective first-line treatment, yielding a mean HbA1c reduction of 1.0-1.5%, reinforcing its position as the foundation of pharmacological therapy for most patients with T2DM [19]. Both SGLT2 and DPP-4 inhibitors showed comparable efficacy in glycemic control, with mean HbA1c reductions ranging between 0.5% and 1.0%, making them valuable alternatives or adjuncts to metformin, particularly for patients needing additional glucose-lowering agents [20,21]. As expected, insulin demonstrated the most potent glucose-lowering effect, especially in patients with more advanced T2DM or those who have exhausted oral therapy options, leading to substantial reductions in HbA1c [22]. However, the sustainability of glycemic control differed among the treatments. Both SGLT2 inhibitors and metformin exhibited more durable effects over time, with consistent long-term control of blood glucose, whereas DPP-4 inhibitors showed a tendency for reduced efficacy over extended periods, with gradual loss of glucose control noted in some cases [23]. These findings highlight the need for careful consideration of treatment longevity, particularly in selecting agents for long-term diabetes management.

One of the most notable findings of our review is the substantial cardiovascular benefit associated with SGLT2 inhibitors, which has been consistently demonstrated across multiple large-scale clinical trials. For instance, the EMPA-REG OUTCOME trial showed a significant 38% reduction in cardiovascular death among high-risk patients with T2DM, while the CANVAS program reported a 14% reduction in major adverse cardiovascular events (MACE), including cardiovascular death, myocardial infarction, and stroke. Similarly, the DECLARE-TIMI 58 trial further reinforced these findings, highlighting a 17% reduction in the risk of cardiovascular death or hospitalization for heart failure. The cardioprotective effects of SGLT2 inhibitors seem to extend beyond glycemic control, suggesting potential benefits through mechanisms such as improved myocardial metabolism, reduced oxidative stress, and favorable hemodynamic effects such as blood pressure and volume reduction. Importantly, this benefit appears to be a class effect, meaning that it is observed across different SGLT2 inhibitors and is particularly pronounced in patients with established cardiovascular disease or multiple risk factors, including heart failure and chronic kidney disease [4,24,25]. These findings underscore the growing recognition of SGLT2 inhibitors not just as glucose-lowering agents but also as pivotal therapies for reducing cardiovascular morbidity and mortality in high-risk populations.

In contrast to the pronounced cardiovascular benefits seen with SGLT2 inhibitors, DPP-4 inhibitors have demonstrated a neutral effect on cardiovascular outcomes. Large clinical trials, including the TECOS, EXAMINE, and SAVOR-TIMI 53 studies, consistently show that DPP-4 inhibitors neither increase nor decrease the risk of MACE, positioning them as a relatively safe option for patients concerned about cardiovascular risks. This cardiovascular neutrality is particularly valuable in patients with T2DM who are at high cardiovascular risk but cannot tolerate other therapies with more profound cardiovascular effects, such as SGLT2 inhibitors or GLP-1 receptor agonists [26]. Meanwhile, metformin, a long-standing first-line therapy, has shown modest cardiovascular benefits, particularly in overweight and obese patients with T2DM. The UKPDS trial provided early evidence of metformin's ability to reduce the risk of myocardial infarction and diabetes-related death in this population, making it a cornerstone of T2DM management for those with concomitant cardiovascular risk factors [27]. On the other hand, the cardiovascular effects of insulin remain more complex and controversial. Some studies, such as the ORIGIN trial, suggest a potential increased risk of cardiovascular events, particularly in patients receiving high doses of insulin or those with advanced disease, likely due to insulin's association with weight gain, hypoglycemia, and potentially adverse metabolic effects, including hyperinsulinemia and endothelial dysfunction [28]. Consequently, the cardiovascular safety of insulin continues to be debated, emphasizing the need for individualized patient management.

SGLT2 inhibitors have emerged as particularly beneficial for renal outcomes. Multiple trials have shown that these agents slow the progression of chronic kidney disease and reduce the risk of end-stage renal disease in patients with T2DM [29]. The renoprotective effects of SGLT2 inhibitors appear to be independent of their glucose-lowering action, suggesting a direct effect on kidney function. DPP-4 inhibitors and metformin have shown neutral effects on renal outcomes [30,31]. While effective in glycemic control, insulin does not appear to offer specific renal protection beyond that conferred by improved glucose control [32].

Weight effects varied significantly among the treatments. SGLT2 inhibitors consistently demonstrated weight loss benefits, with average reductions of 2-3 kg over 6-12 months [33]. This weight loss effect is attributed to both caloric loss through glucosuria and a shift in substrate utilization towards lipid oxidation. DPP-4 inhibitors are generally weight-neutral [34], while metformin may lead to modest weight loss or weight neutrality [35]. Insulin therapy, however, is often associated with weight gain, which can be a significant concern for many patients with T2DM [36].

The risk of hypoglycemia varied among the treatments, with important implications for patient safety and quality of life. SGLT2 inhibitors and DPP-4 inhibitors demonstrated a low risk of hypoglycemia when used as monotherapy or in combination with metformin [37,38]. Metformin also carries a low risk of hypoglycemia [39]. In contrast, insulin therapy is associated with a significantly higher risk of hypoglycemia, particularly in intensive treatment regimens [40].

While our review offers valuable insights, several limitations should be recognized. First, the significant heterogeneity in study designs, populations, and outcome measures among the included trials may restrict the ability to make direct, meaningful comparisons across studies. This variability complicates the interpretation of results as the studies assess different subgroups of patients with varying baseline characteristics, which could influence the treatment outcomes. Second, most of the included studies had relatively short follow-up periods, typically ranging from one to five years, which may not be sufficient to capture the long-term effects and safety profiles of the medications. This highlights the need for longer-term studies to better evaluate the durability of treatment effects, particularly in chronic conditions such as T2DM. Future research should prioritize head-to-head trials comparing these medication classes in diverse patient populations, including those with differing comorbidities, to establish more definitive evidence of comparative efficacy. Moreover, exploring the optimal sequencing and combination of these therapies is crucial to developing personalized treatment strategies that maximize clinical benefits while minimizing risks. These studies should also consider patient preferences, quality of life, and cost-effectiveness to inform more tailored, patient-centered care approaches.

Our systematic review highlights the distinct efficacy and safety profiles of SGLT2 inhibitors, DPP-4 inhibitors, metformin, and insulin in the management of T2DM. While all agents effectively improve glycemic control, SGLT2 inhibitors stand out for their cardiovascular and renal benefits. Metformin remains a valuable first-line option, particularly given its long-term safety data and low cost. DPP-4 inhibitors offer a well-tolerated alternative with neutral effects on weight and cardiovascular outcomes. Insulin remains the most potent glucose-lowering agent but carries increased risks of hypoglycemia and weight gain. These findings underscore the importance of a patient-centered approach to T2DM management, considering individual patient characteristics, comorbidities, and treatment goals. As our understanding of these medications continues to evolve, ongoing research will be crucial in refining treatment algorithms and improving outcomes for patients with T2DM.

## Conclusions

This systematic review highlights the distinct profiles of SGLT2 inhibitors, DPP-4 inhibitors, metformin, and insulin in the management of T2DM, offering valuable insights into their comparative efficacy and long-term outcomes. While all four therapies are effective in glycemic control, SGLT2 inhibitors stand out for their significant cardiovascular and renal benefits, making them particularly advantageous for patients with comorbid conditions. Metformin remains a cost-effective first-line option with a solid safety profile, while DPP-4 inhibitors provide a well-tolerated alternative with neutral effects on weight. Insulin, although the most potent glucose-lowering agent, is associated with higher risks of hypoglycemia and weight gain. These findings underscore the need for individualized treatment approaches in T2DM, taking into account each patient's unique clinical profile and therapeutic goals. Ongoing research is essential to further refine treatment strategies and optimize outcomes in diverse patient populations.
